# Native Macrophyte Density and Richness Affect the Invasiveness of a Tropical Poaceae Species

**DOI:** 10.1371/journal.pone.0060004

**Published:** 2013-03-25

**Authors:** Thaisa S. Michelan, Sidinei M. Thomaz, Luis M. Bini

**Affiliations:** 1 Departamento de Biologia, Universidade Estadual de Maringá, Nupelia, Maringá, PR, Brazil; 2 Departamento de Ecologia, Universidade Federal de Goiás, ICB, Campus II, Goiânia, GO, Brazil; Estacion Experimental de Zonas Áridas (CSIC), Spain

## Abstract

The role of the native species richness and density in ecosystem invasibility is a matter of concern for both ecologists and managers. We tested the hypothesis that the invasiveness of *Urochloa arrecta* (non-native in the Neotropics) is negatively affected by the species richness and abundance of native aquatic macrophytes in freshwater ecosystems. We first created four levels of macrophyte richness in a greenhouse (richness experiment), and we then manipulated the densities of the same native species in a second experiment (density experiment). When the native macrophytes were adults, fragments of *U. arrecta* were added, and their growth was assessed. Our results from the richness experiment corroborated the hypothesis of a negative relationship between the native species richness and the growth of *U. arrecta*, as measured by sprout length and root biomass. However, the resistance to invasion was not attributed to the presence of a particular native species with a greater competitive ability. In the density experiment, *U. arrecta* growth decreased significantly with an increased density of all five of the native species. Density strongly affected the performance of the Poaceae in a negative manner, suggesting that patches that are densely colonized by native macrophytes and less subject to disturbances will be more resistant to invasion than those that are poorly colonized and more commonly subjected to disturbances. Our density experiment also showed that some species exhibit a higher competitive ability than others (sampling effect). Although native richness and abundance clearly limit the colonization and establishment of *U. arrecta*, these factors cannot completely prevent the invasion of aquatic ecosystems by this Poaceae species.

## Introduction

Biological invasions have been the focus of a number of ecological investigations [1] because they are one of the most significant causes of global ecological change [2]. Such invasions represent a threat to local biodiversity in both terrestrial and aquatic ecosystems, and they are considered one of the main causes of extinctions [3]. Naturalists have long postulated that species invasiveness may be negatively affected by native species richness. Indeed, Darwin [4] was one of the first to propose that species-rich communities are less prone to invasion than species-poor communities. This hypothesis was revisited by Elton [5], whose ideas have come to constitute the core of the invasion debate during the last five decades [6], [7]. The hypothesis that communities containing a greater number of species are less invasible (biotic resistance) has since been addressed in several investigations [8]. Most of the obtained results have shown that although this hypothesis holds at small spatial scales [1], [9]–[10], it is frequently uncorroborated at large spatial scales, where the relationship between invasion success and native richness becomes positive [11]–[15]. However, the majority of these investigations were conducted in terrestrial ecosystems, and one can therefore argue about the generality of the *diversity - invasibility* hypothesis.

Niche partitioning is often considered to be a mechanism that explains biotic resistance. According to this hypothesis, the greater the number of species in a community, the greater the use of different resources and biomass production, leading species to become complementary in space and time [16]. Thus, native species complement each other in terms of resource use in a given habitat, competing with non-native species and reducing the latter group's success. The alternative hypothesis to niche complementarity is that biotic resistance may result from the presence of a particular native species with a high competitive ability, which reduces the invasiveness of non-native species [11], [17]. Thus, the biotic resistance to invasion found in species-rich communities could be explained by the increased chance of finding one such highly competitive species (constituting a *sampling effect* [1]).

In addition to diversity, the native species abundance may negatively affect ecosystem invasibility by non-native species [13]–[14], [18]. Tests of this hypothesis in observational studies conducted in northern temperate lakes have provided contrasting results, as for instance, some species of macrophytes respond negatively to an increased abundance of natives, whereas other species remain unaffected [14].

The high biodiversity of inland water bodies can be partially attributed to the presence of aquatic macrophytes [19]. Given the importance of these macrophytes, the causes and potential impacts of invasion by non-native macrophyte species have become central issues in studies of aquatic ecosystem invasion. *Urochloa arrecta* (Hack. ex T. Durand & Schinz) Morrone & Zuloaga is known to have invaded several natural and artificial aquatic ecosystems in South America [20], resulting in a negative impact on native aquatic communities [21]–[22]. This member of the Poaceae family (described as *Urochloa subquadripara* by Thomaz et al [15], Michelan et al [22] and Michelan et al. [23]) is highly invasive because it regenerates readily from both fragments [23] and seeds [24] and exhibits an efficient dispersion strategy, high relative growth rates and rapid recovery rates after disturbances [15], [23].

A large-scale observational study showed that *Urochloa arrecta* invaded sites (*ca*. 100 m long patches of macrophytes) with higher numbers of native species more successfully, suggesting that its invasion potential is regulated by abiotic factors that also affect native species [15]. In contrast, the occurrence of *U. arrecta* in 1 m^2^ plots was observed to decrease with increasing native richness [22]. This apparent contradiction (i.e., opposite responses of invasibility at different spatial scales) has been found elsewhere [14], [25], and thus, experiments assessing the effects of biotic or abiotic factors on *U. arrecta* invasiveness might be useful.

In this study, we experimentally evaluated the effect of native macrophyte species richness and abundance on the invasive potential of *Urochloa arrecta*. We tested the hypothesis that the invasibility of freshwater environments by *U. arrecta* is negatively related to the richness and abundance of native species. The rationale for this hypothesis is that at small scales, competition is expected between native and non-native species, negatively impacting the latter group [6]. While we predicted that *Urochloa arrecta* growth would decrease with increasing native richness and biomass, we also anticipated that the strength of these (negative) relationships would increase as the values of native richness and/or native biomass increased. In addition, by comparing the effects of different monocultures on *U. arrecta* growth, we were able to test for sampling effects.

## Materials and Methods

### Species selection

In 2010, two experiments were conducted in a greenhouse at the University of Maringá in Brazil using five native species that co-occur with *U. arrecta* at high (55-35%), intermediate (16-9%) and low (1%) frequencies. These frequencies were estimated based on the proportion of samples containing both the native species and *U. arrecta* (see next paragraph). The species were selected based on different levels of co-occurrence with *U. arrecta* because choosing only native species that frequently co-occur with a non-native species could bias the experiment by favoring natives that are less resistant to invasion. Similarly, choosing only native species that co-occur less frequently with a non-native species could favor species that are highly resistant to invasion.

To evaluate the patterns of co-occurrence, we used a dataset comprised of 1,404 samples (combination of 117 sites×12 monitoring times) collected in the Itaipu Reservoir (Brazil) from 2005–2007. Twenty-five emergent species were found to co-occur with *U. arrecta* in this freshwater ecosystem; using the criteria outlined above, the following species were selected: *Panicum pernambucense* (Spreng.) Mez ex Pilg. and *Leersia hexandra* Sw. (two native Poaceae species often found to co-occur with *U. arrecta*: 55-35%), the Cyperaceae *Eleocharis montana* (Kunth) Roem. and Schult. and the Commelinaceae *Commelina diffusa* Burm. f. (both showing an intermediate level of co-occurrence: 16-9%) and finally, the Pontederiaceae *Pontederia cordata* L. (presenting a low level of co-occurrence: ≤1%). Together with *U. arrecta*, specimens of the above-mentioned native species were obtained from streams, rivers and reservoirs in the Upper Paraná River basin. The sediment used in the experiments was collected from a reservoir at a site dominated by *U. arrecta*. During the experiment, the trays were inspected daily, and the buds of other species were removed.

### Experiment 1 – Effect of species richness and abundance on the U. arrecta invasion potential (poly- and monocultures)

To test the effects of native species richness on *U. arrecta* invasiveness, we used trays (0.70×0.45×0.30 m, with a 3–5 cm-deep layer of water) in which four different levels of richness were generated, corresponding to 0, 1, 3 or 5 species. All possible combinations of species within each level were included (see Figure S1 in the Supporting Information), resulting in a total of 60 trays: five with no species (zero richness level), 15 with one species (five monocultures×three replicates), 30 with three species (10 combinations×three replicates) and 10 with five species (one combination×10 replicates). The total density of native species was kept constant (15 individuals per tray, corresponding to 47 plants m^−2^). Thus, the trays containing three species contained five individuals of each species, whereas those with five species contained three individuals of each species.

The selected native species are characterized by short lifecycles. Therefore, after 120 days, all individuals were adults (all were flowering, with the exception of *P. pernambucense*). We then added eight apical fragments of *U. arrecta* (with two nodes each) to each tray. After 75 days, all of the plants (both native and invasive species) were removed, then separated by species and oven-dried at 70°C to a constant weight. As a measure of *U. arrecta* growth, we quantified the following traits: sprout length and aboveground and root dry weight biomass. For each native species, we measured the dry weight biomass of aboveground tissues and roots.

### Experiment 2 – Effect of species abundance on the U. arrecta invasion potential (monocultures)

To test the effect of native species abundances alone on *U. arrecta* invasiveness, one of six density levels for each of the native species (monocultures with 0, 1, 3, 5, 7 and 10 individuals, corresponding to 0, 14, 41, 69, 96 and 138 plants m^−2^, respectively) was assigned to individual trays (0.33×0.22×0.10 m, with a 3–5 cm-deep layer of water). Three replicates for each species and 10 replicates for the control group (with an absence of native plants) were performed, totaling 85 trays. As in experiment 1, after 120 days, we added four apical fragments of the non-native species to each tray. After 75 days, all plants were removed and oven-dried at 70°C to a constant weight. We measured the same traits here as in experiment 1.

Because native species intercept light and potentially interfere with *U. arrecta* growth, photosynthetically active radiation (PAR) levels were measured directly above and underneath (close to the microcosm sediment) plant leaves using a PAR meter (Li-Cor: model Li-182). The percentage of PAR (% PAR) retained by the vegetation was then estimated.

We emphasize that the two experiments were conducted independently. Thus, in the first experiment, among-species synergistic effects could have occurred because several species were interacting with each other, while in the second experiment, the use of single species prevented among-species synergistic effects. Therefore, we could test for the effect of richness and the associated potential among-species synergies (Experiment 1) and then test separately for the effect of abundance freely of confounding synergistic effects (Experiment 2).

### Statistical analyses

The effects of native species richness and biomass on *U. arrecta* growth (Experiment 1 – poly – and monocultures) were tested via multiple regressions. The mean values of the length and aboveground and root biomass of *U. arrecta* plants were used as response variables, and the richness and aboveground and root biomasses of native species were used as explanatory variables. All first-order interactions between the explanatory variables were included in the models to test for multiplicative effects. Prior to these analyses, we used the Box–Cox transformation to correct for departures from normality. The Shapiro-Wilk (W) test was performed to check the residuals for normality. A significant negative effect of native richness alone - and not of the identities of individual species (a *sampling effect*, see next paragraph) - would favor the hypothesis that relates the invasiveness potential to native diversity (i.e., biotic resistance). Similarly, significant and isolated effects of the native aboveground or root biomass would indicate the influence of competition for light or nutrients, respectively. These effects would more likely be related to interspecific competition, as a density-dependent process, than either the effect of richness *per se* or diffuse competition [26].

Some investigations have shown that resistance to non-native species invasions can be the result of particular native species that may be regarded as superior competitors (a *sampling effect* [11], [27]). To assess whether a particular native species showed a superior competitive ability over *U. arrecta*, a multivariate analysis of variance (MANOVA) was conducted, considering the five native species used in the monocultures as a classification factor and the variables indicative of *U. arrecta* growth (i.e., the mean length and aboveground and root biomass) as response variables. Differences in *U. arrecta* traits between the monocultures would suggest that native species offer different degrees of resistance to invasion and, thus, would indicate that a sampling effect had occurred.

The effects of density and native species identity on *U. arrecta* growth (Experiment 2 – monocultures) were tested via an analysis of covariance (ANCOVA). We used the native densities and the aboveground and root biomass as predictors. For each response variable (mean length and aboveground and root biomass), the ANCOVA model considered native density to be a continuous variable and plant identity to be a categorical factor. This analysis tested the hypothesis of parallelism, that is, whether the effect of density (continuous predictor) was similar for all five native species (categorical predictor). Considering that in the control group, there were no native individuals, the values of the response variables obtained under these conditions were distributed randomly among all groups formed by native species [28]. We also conducted an ANCOVA to test the effects of species density and species identity on the % PAR. All analyses were conducted using the R environment for statistical computing [29].

### Comparison with meta-analytical results

Finally, we compared our results with those obtained in previous meta-analyses to assess whether the effect sizes of biotic resistance (the direction of the comparison between groups: the performance of a non-native species without biotic resistance minus its performance with biotic resistance) detected in our study were similar to those observed elsewhere. The details of this analysis are provided in Appendix S1 in the Supporting Information.

## Results

### Experiment 1 – Effect of species richness and abundance on the U. arrecta invasion potential (poly- and monocultures)

According to the adjusted coefficient of determination (Adj. *R* ^2^) derived from the multiple regression analysis, the explanatory variables accounted for 44.8% of total variation in *U. arrecta* length (*F*_6,52_ = 8.85, *P*<0.001). In addition to the negative and significant effect of native species richness on *U. arrecta* length, a positive and significant interaction between the native aboveground and root biomasses was detected (Table 1 and Figure S2 in the Supporting Information).

**Table 1:** Partial regression coefficients (± standard errors) derived from multiple regression models evaluating the relationship between *U. arrecta* traits and three explanatory variables.

Response variable	Explanatory variables	Estimate	*SE*	*t*	*P* (>|*t*|)	Adj. *R* ^2^
Sprout length	(Intercept)	61.442	1.60115	38.37	0.000	0.45
	Native species richness (1)	−2.68217	1.06193	−2.53	0.015	
	Native aboveground biomass (2)	−0.00861	0.03642	−0.24	0.814	
	Native root biomass (3)	−0.23004	0.06369	−3.61	0.001	
	(1)×(2)	0.00512	0.01334	0.38	0.703	
	(1)×(3)	0.03203	0.01781	1.80	0.078	
	(2)×(3)	0.00082	0.00029	2.81	0.007	
Aboveground biomass	(Intercept)	−0.048	0.05209	−0.92	0.363	0.47
	Native species richness (1)	0.01545	0.03455	0.45	0.657	
	Native aboveground biomass (2)	−0.00252	0.00119	−2.13	0.038	
	Native root biomass (3)	−0.00206	0.00207	−0.99	0.325	
	(1)×(2)	−0.00028	0.00043	−0.65	0.520	
	(1)×(3)	−0.00001	0.00058	−0.02	0.983	
	(2)×(3)	0.00002	0.00001	1.61	0.114	
Root biomass	(Intercept)	−0.009	0.16080	−0.06	0.954	0.16
	Native species richness (1)	−0.24470	0.10670	−2.29	0.026	
	Native aboveground biomass (2)	0.00076	0.00366	0.21	0.835	
	Native root biomass (3)	−0.00728	0.00640	−1.14	0.260	
	(1)×(2)	0.00177	0.00134	1.32	0.193	
	(1)×(3)	0.00123	0.00179	0.69	0.496	
	(2)×(3)	0.00001	0.00003	0.44	0.664	

The explanatory variables accounted for 46.8% of the variance in the *U. arrecta* aboveground biomass (*F*_6,52_ = 9.49, *P*<0.001), and the *t*-tests associated with the partial regression coefficients indicated that only the native aboveground biomass had a significant and negative effect (Table 1). The *U. arrecta* root biomass was significantly and negatively related to the native species richness (*F*_6,52_ = 2.85, *P* = 0.018, Adj. *R* ^2^ = 16.1%; Table 1). In all cases, the assumption of normality of the residuals was satisfied. All results described above were obtained after the exclusion of a large outlier (detected by Cook's distance). However, the results were qualitatively similar when this outlier was not removed.

**Figure 1:**
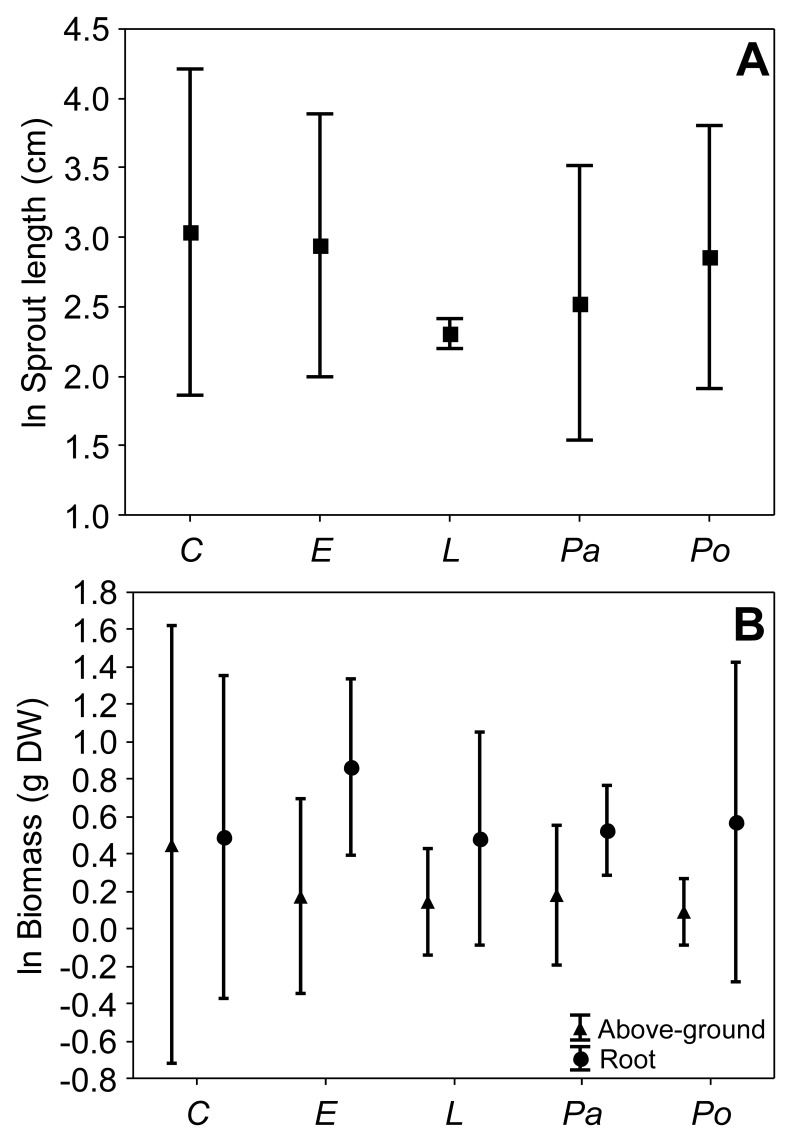
Effects of single native species on *Urochloa arrecta* growth. Sprout length (A) and aboveground and root biomass (B) of *U. arrecta* (mean ± 95% CI) when cultivated in monocultures in our first experiment. *C* = *Commelina diffusa*, *E* = *Eleocharis montana*, *L* = *Leersia hexandra*, *Pa* = *Panicum pernambucense*, *Po* = *Pontederia cordata*.

According to a MANOVA using the *U. arrecta* length and the aboveground and root biomass as response variables, the native monocultures did not significantly affect *U. arrecta* growth (Wilk's Lambda = 0.217, *P* = 0.242; Figure 1). These results indicate that in the experiments assessing the effects of species richness, the effect of individual native species on *U. arrecta* growth did not vary.

### Experiment 2 – Effect of species abundance on the U. arrecta invasion potential (only monocultures)

**Figure 2:**
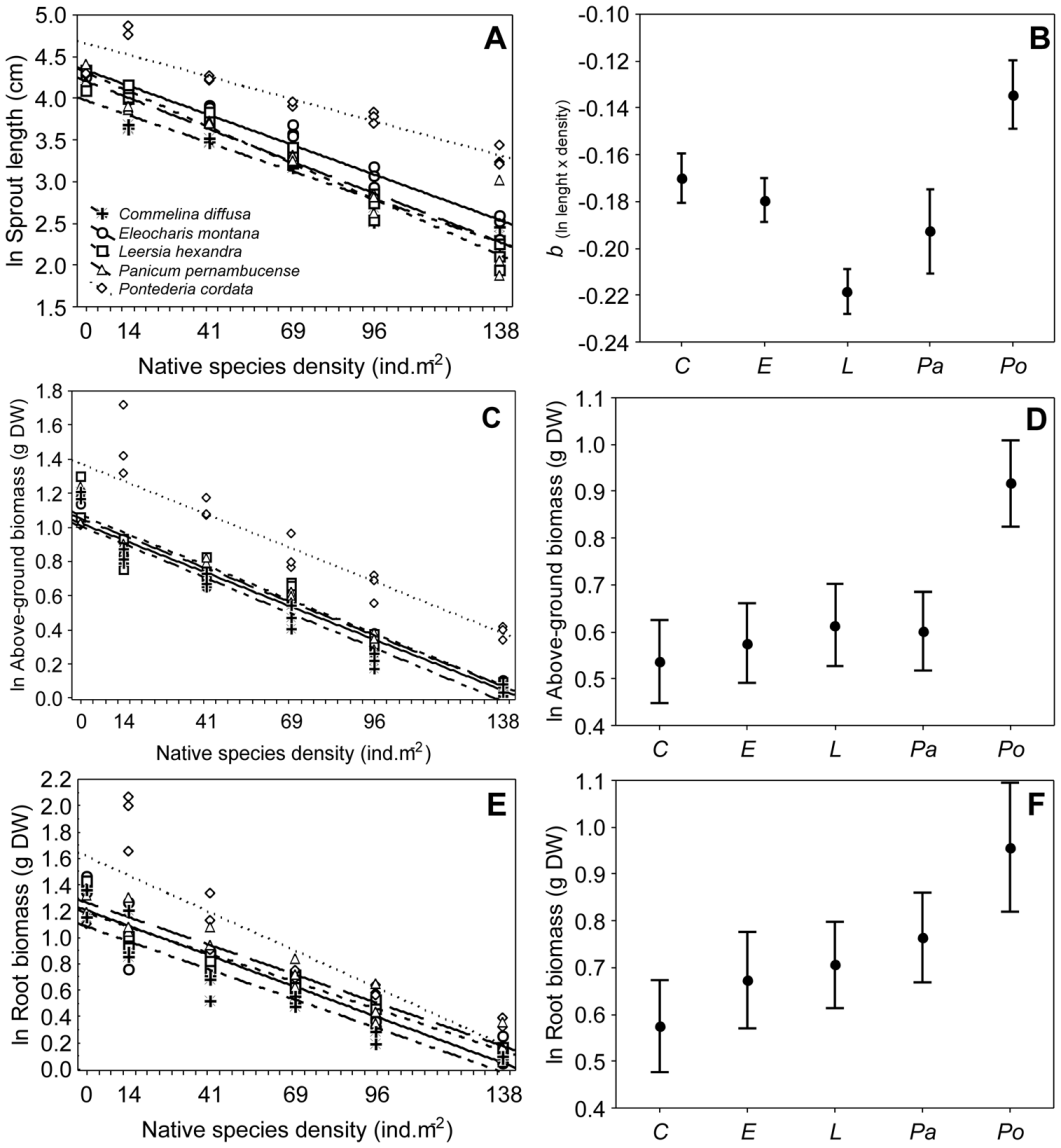
Effects of the densities of five native species on the exotic species. Relationships between native species densities and *Urochloa arrecta* sprout length (A) and aboveground (C) and root (E) biomass. The slopes of the relationships between native density and *U. arrecta* length (B) and the adjusted means (± SE) of aboveground (D) and root biomass (F) are also shown. The values shown in A, C and E represent the means for each tray obtained from the monocultures used in our second experiment.

*Urochloa arrecta* length was strongly and negatively affected by the native species density, although this effect differed among species (Figure 2A), as indicated by the significant interaction between species identity and density (ANCOVA, *F*_4,75_ = 5.8, *P*<0.01). *Leersia hexandra* and *P. cordata* showed the largest and smallest negative effects, respectively, on *U. arrecta* length (Figure 2B). In contrast, the hypothesis of parallelism (i.e., equal slopes) for the aboveground biomass (Figure 2C) and root biomass (Figure 2E) was not rejected (ANCOVA, *F*_4,75_ = 1.7, *P* = 0.167 and *F*_4,75_ = 0.8, *P* = 0.515, respectively). These results indicate that the negative effect of density on *U. arrecta* growth (in terms of slopes) did not differ among the five native species. The adjusted mean values revealed a significantly higher *U. arrecta* aboveground biomass (effect of species, *F*_4,79_ = 16.1, *P*<0.01) and root biomass (effect of species, *F*_4,79_ = 8.23, *P*<0.01) when individuals of this species were cultivated with *P. cordata*. The lowest values of *U. arrecta* biomass, in terms of both the aboveground parts and roots, were observed when this species grew in conjunction with *C. difussa* or *E. montana* (Figs. 2D and 2F). These two native species are the species that co-occurred at intermediate levels with *U. arrecta* in the field. Thus, in contrast to the richness experiment, the density experiment indicated that a sampling effect might explain *U. arrecta* invasiveness.

In general, the comparisons between the coefficients of determination estimated in both experiments suggested that the native species density was likely to be a better predictor of *U. arrecta* growth than was native richness (for models including this predictor, see coefficients of determination above: results from experiment 1) with respect to plant length (ANCOVA; *F*_1,75_ = 966.3, *R* ^2^ = 0.94, *P*<0.01), aboveground biomass (*F*_1,79_ = 675.2, *R* ^2^ = 0.90, *P*<0.01) and root biomass (*F*_1,79_ = 483.29, *R* ^2^ = 0.87, *P*<0.01).

The native species biomass also negatively affected *U. arrecta* attributes (results not shown). Similar to the results obtained using native species densities as explanatory variables, *C. diffusa* showed the greatest effect on *U. arrecta* growth, and *P. cordata* had the smallest effect (see Figure S2). However, the biomass data showed no linear tendencies, even after being log transformed. Therefore, an ANCOVA was not applied to these results.

**Figure 3:**
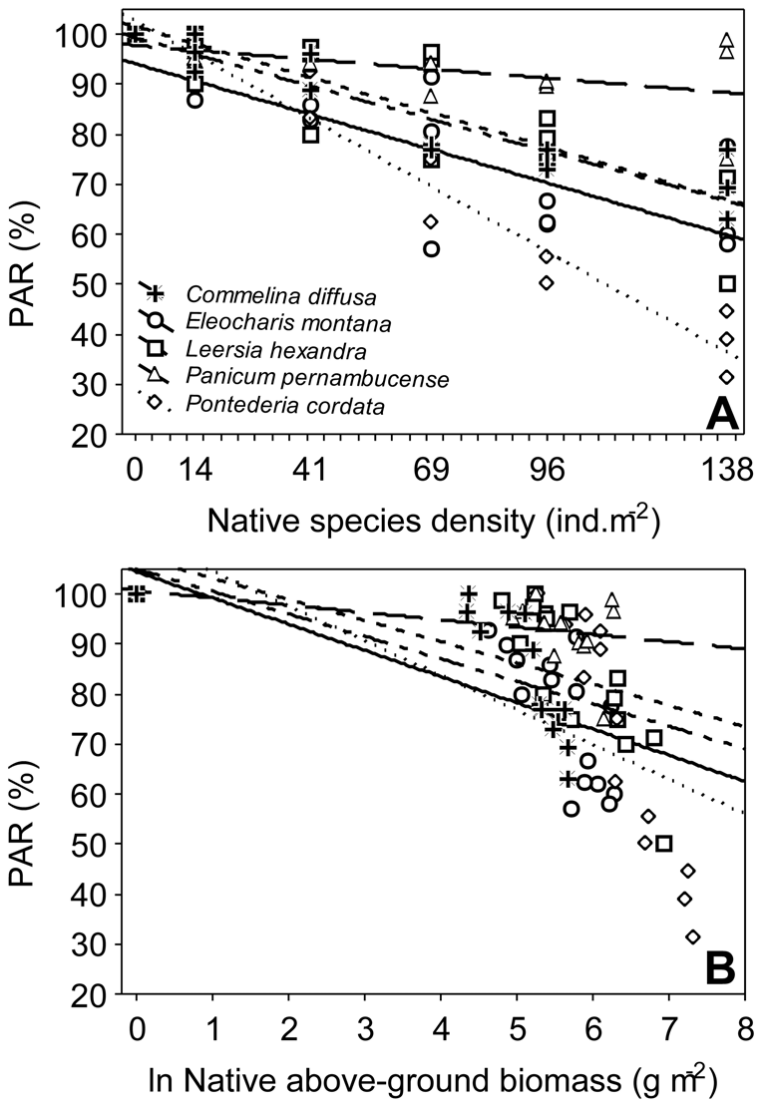
Effects of the native macrophytes on photosynthetic active radiation (PAR %). Relationship between plant density (A) and aboveground biomass (B) and PAR reaching the sediment surface.

An increase in the native species density significantly reduced the % PAR reaching the sediment (*R* ^2^ = 0.81, *P*<0.01); however, the extent of this effect differed among the five species (Figure 3A), as indicated by the significant interaction between plant identity and plant density (ANCOVA, *F*_4,65_ = 13.85, *P*<0.01). *Pontederia cordata* and *P. pernambucense* showed the largest (*b* = −0.066±0.004) and smallest (*b* = −0.009±0.004) negative effects, respectively, on the % PAR reaching the sediment (Figure 3A). A significant interaction was also observed when the aboveground biomass of native species was used as a predictor variable (ANCOVA, *F*_4,65_ = 8.09, *P*<0.01; Figure 3B). As seen for the species density, the aboveground biomass of *P. cordata* and *P. pernambucense* had the largest and smallest effects, respectively, on the % PAR (*b* = −0.128±0.03 and −0.027±0.01, Figure 3B).

### Comparison with meta-analytical results

The accumulated effect size (comparing two groups: *U. arrecta* growing alone and in association with natives) based on the Hedges' statistic (*d*_++_ = 1.72) was statistically significant (CI_95%_ = 1.08 and 2.26) and was similar to that estimated by Levine et al. [8] (see Figure S3 in Supporting Information). The heterogeneity of the effect sizes calculated for the five native species was not significant (*Q* = 4.8, g.l. = 4, *P* = 0.311).

The value of Fisher's *z* statistic (based on transformation of Pearson's correlation coefficient) was −0.49 for the relationship between the species richness and *U. arrecta* length and −0.36 for the relationship between the native species richness and total *U. arrecta* biomass. Both values were similar to those obtained by Balvanera et al. [30].

Finally, similar to the results calculated for Hedges' *d* statistic, the log response ratios were −0.88 (variance = 0.009) for the mean length and −0.67 (variance = 0.01) for the total biomass. These effect sizes indicate that *U. arrecta* growth was lower when this species was cultivated in a polyculture consisting of five native species. Similar results were found by Cardinale et al. [31].

## Discussion

Our results are in line with the hypothesis that the growth of non-native species is negatively affected by the richness and abundance of native species. Other researchers have also found that the native species richness and abundance negatively affect non-native invasiveness at small spatial scales [1], [9], [13]–[14]. However, the findings of the present study offer a new insight, as we used the same group of species to independently test the effectsof richness and abundance.

The decrease in *U. arrecta* length and root biomass observed with an increase of native richness in experiment 1 (*richness experiment*) likely reflects complementarity among all native species [16], rather than a sampling effect. We discard a potential sampling effect because *U. arrecta* growth did not differ among the individual native species (see Figure 2). According to the complementarity mechanism, available resources are used more thoroughly at sites with a high native species richness, reducing non-native success [27]. In contrast, the sampling effect scenario predicts that species-rich communities have a greater chance of including a highly competitive native species, which reduces non-native invasiveness [11], [17]. Thus, according to the sampling effect, the decrease in non-native invasiveness would not be explained by native species richness *per se*, but by competition between the non-native species and a particular native species.

We also detected a significant interaction between the aboveground and root biomasses of native species that affected *U. arrecta* length. Specifically, the slope of the relationship between the fitted values of *U. arrecta* length and the aboveground biomass of the native species changed from almost null to positive with increasing values of the root biomass of the native species (Figure S2 in Supporting Information). Although this result is counterintuitive and actually contrary to our prediction, it is in accordance with results obtained in some experimental studies designed to test the relative importance of aboveground and belowground competition (e.g., [32] and references therein). We speculate that the rates of shoot elongation in response to increased levels of aboveground competition occur only at high levels of belowground competition. However, this result mainly highlights the complexity of competitive interactions in plants.

In addition to richness, plant density has been considered an important variable for explaining invasion success. Indeed, the effect of density on invasion success has been demonstrated, especially in the field [14] but also in experiments testing competition between native and non-native species [33]–[34]. Despite some uncertainties regarding the relative importance of sampling and complementarity effects, interpreting the results of our two experiments in conjunction allows us to infer that the density of any of the native species was a more important factor in explaining the *U. arrecta* invasion success than was native richness. We based this inference on the weak effect of species richness observed in experiment 1 (see Table 1 and the low coefficients of determination) and on the sharp decrease in *U. arrecta* biomass with increasing native densities observed in experiment 2 (see Figure 2). However, our inference of the greater importance of density, compared to richness, should be viewed with caution because (i) our experiments were conducted separately (one including the effect of species richness plus biomass and the other only addressing monoculture biomass), and (ii) we manipulated a small number of native species (a maximum of five). To disentangle these effects in a more conclusive way, future studies should adopt a full factorial design to test the effects of richness, density and their interaction, despite the inherent difficulty in terms of the large sample size that would be required for this task.

Macrophytes are usually found in patches dominated by one or several species with a similar growth form [35], and the biomasses found in tropical lakes (e.g., [36]) are of the same order of magnitude s those recorded in our highest density treatments (see Figure S2). Thus, under field conditions, we expect that *U. arrecta* fragments face approximately the same competitive pressure encountered at the higher levels of densities involved in our second experiment, suggesting that under such conditions, invasibility is reduced compared to patches with a lower native biomass.

Based on the results of our second experiment, we can speculate about the type of competitive interaction that occurs between *U. arrecta* and native species. For example, we found that an increase in the density of *L. hexandra* had the highest negative impact on *U. arrecta* length. *L. hexandra* could compete with *U. arrecta* for light in littoral habitats because both species have floating stems. Still, according to our second experiment, the effect of the native species on *U. arrecta* length differed along the gradient of native biomass, as indicated by the significant interaction term (Fig. 2A). Thus, it appears that at least for this trait, there is a sampling effect that is dependent on native biomass. Plant elongation is mainly affected by light availability [37], and differences in light interception among native species explain our results because a significant interaction term for the % PAR reaching sediment was also found (Fig. 3A).

In our second experiment, *C. diffusa* and *E. montana* were the species that had the greatest effect on the *U. arrecta* aboveground biomass, largely via inhibiting its establishment (i.e., root biomass). Our results suggest that these native emergent species could compete more efficiently with *U. arrecta* for space and nutrients during the early stages of invasion, as simulated in our experiment. In addition, the intensity of root competition may be strongly related to plant density [38]–[39], as suggested by the reduction of *U. arrecta* length and above ground biomass and root biomass detected in response to increases in the native species density and biomass. Thus, the results of the second experiment (Figs. 3D and 3F), where we manipulated a greater variety of native biomasses than in the first experiment, suggest that *C. diffusa* and *E. montana* might exert a stronger invasion resistance effect than the other native species. These results could partially explain why these two species are found in only 9–16% of the sites colonized by *U. arrecta* in the field (see Methods). The fastest growth of *U. arrecta* was found when this species was cultivated with *P. cordata* (Figs. 3B, 3D and 3F), indicating that *P. cordata* offers the lowest resistance to invasion compared to the other four native species. Thus, patches colonized only by *P. cordata* may represent sites where invasion by *U. arrecta* succeeds more rapidly in freshwater ecosystems.

In general, an increase in the native species density and biomass reduces PAR close to sediment, which in turn, decreases the success of non-native species [37]. Although a reduction of PAR (see Figure 3) can partially explain the decrease in the *U. arrecta* invasiveness potential, our results indicate that this effect was secondary because the smallest negative effect on *U. arrecta* success was caused by *Pontederia cordata*, which affected light intensity more than any of the other native species. This finding reinforces the hypothesis that other density-dependent mechanisms, such as competition for space and/or nutrients, are more important than light attenuation. However, *Urochloa arrecta* showed the greatest elongation when grown with *P. cordata*, which is in accordance with the findings of studies on both terrestrial (e.g., [37]) and aquatic macrophytes (e.g., [40]–[41]) showing that non-native plants growing under light-limiting conditions exhibit increased elongation.

Our results also allow some inferences to be made about the relationships between *U. arrecta* invasiveness and the disturbance regimes in freshwater ecosystems. Studies comparing the growth of native and non-native macrophytes have shown that the latter are usually favored after disturbance events [42]–[43]. Disturbed sites generally exhibit a low native species richness and abundance, which may favor *U. arrecta* growth, as suggested by our results. In addition, natural and anthropogenically induced droughts are common disturbances in several types of freshwater ecosystems. *Urochloa arrecta* is highly resilient to drought, and it can regenerate, even after 26 days of desiccation [23]. Therefore, *U. arrecta* may invade sites subjected to disturbances more successfully, especially those caused by droughts. In fact, previous studies have further suggested that disturbed aquatic ecosystems are more susceptible to invasion [44]–[45].

Comparison of experimental and meta-analytical results is an interesting way to assess the effect sizes estimated by a given study and to evaluate whether predictions remain valid after considering the results of a specific experiment (e.g., whether the growth of an invasive species is poorer in habitats with a higher native richness and density). These comparisons also enable us to place the results of a specific experiment in a broader context. The accumulated effect sizes found in our study were similar to (or even greater than) those observed by other researchers investigating the diversity-invasibility relationship [8], [30]. The effect sizes are also in line with the main trends found in the literature, specifically that non-native species exhibit a low success where native richness and density are relatively high. Finally, considering that resistance to invasion is an ecosystem function, the effect size recorded in our study was higher than the accumulated effect size found by Cardinale et al. [31]. From these results, we can conclude that the effect sizes estimated in our study are in line with the main trends found in the literature, specifically that non-native species exhibit a low success where native richness and density are relatively high.

In summary, our data support the hypothesis that the invasibility of aquatic ecosystems by *U. arrecta* is negatively affected by native species richness, mainly in terms of native macrophyte abundances. Taking the results of both experiments, we infer that a complex combination of density, richness, complementarity and sampling effects may have occurred in the freshwater ecosystems that we simulated. However, the observed decrease in *U. arrecta* growth in the experimental units with the lowest levels of richness or density indicates that the presence of a single native species, even in small numbers, reduces the invasiveness of this member of the Poaceae. Although the richness and abundance of native species significantly reduced *U. arrecta* growth, our results did not support the view that native species are able to preclude the invasion process, a conclusion that was also reached by Levine et al. [8]. Thus, despite limiting *U. arrecta* success, the effects of native species richness and abundance were not sufficient to completely prevent invasion by this species.

## Supporting Information

Figure S1 **Scheme representing the combination of different native species used in the experiment to assess the effect of native richness on the invasiveness of** ***U. arrecta*** **.**
(DOC)

Figure S2 **Relationships between exotic sprout length (cm, Box-Cox transformed; lambda = −0.31) and the native aboveground biomass, setting the native root biomass to three values. The estimated effects obtained with the package** ***effects*** **are shown** [**1**], [**2**] **.**
(DOCX)

Figure S3 **Accumulated effect sizes (mean ± CI_95%_) from Levine et al. (2004) and from our experiment.**
(DOC)

Appendix S1 **Meta-analysis.**
(DOC)
