# Supplementary material for: Native Macrophyte Density and Richness Affect the Invasiveness of a Tropical Poaceae Species
Source: PLoS One. 2013 Mar 25;8(3):e60004. doi: 10.1371/journal.pone.0060004 (PMC3607602; doi:10.1371/journal.pone.0060004)
Supplement: Figure S1 — Scheme representing the combination of different native species used in the experiment to assess the effect of native richness on the invasiveness of U. arrecta . (DOC) [file pone.0060004.s001.doc]

**Supporting Information**

**Figure S1 –** Scheme representing the combination of different native species used in the experiment to assess the effect of native richness on the invasiveness of tropical *Urochloa arrecta*. C: *Commelina diffusa*, Po: *Pontederia cordata*, Pa: *Panicum pernambucense*, L: *Leersia hexandra*, E: *Eleocharis montana*.

1

**C**

**Po**

**Pa**

**L**

**E**

3

**CPoPa**

**CPoL**

**CPoE**

**CPaL**

**CPaE**

**CL E**

**Po PaL**

**Po PaE**

**PoLE**

**PaLE**

5

CPoPaLE
