# Supplementary material for: Native Macrophyte Density and Richness Affect the Invasiveness of a Tropical Poaceae Species
Source: PLoS One. 2013 Mar 25;8(3):e60004. doi: 10.1371/journal.pone.0060004 (PMC3607602; doi:10.1371/journal.pone.0060004)
Supplement: Figure S2 — Relationships between exotic sprout length (cm, Box-Cox transformed; lambda = −0.31) and the native aboveground biomass, setting the native root biomass to three values. The estimated effects obtained with the package effects are shown [1], [2] . (DOCX) [file pone.0060004.s002.docx]

**Supporting Information**

**Figure S2. Relationships between exotic sprout length (cm, Box-Cox transformed; lambda = -0.31) and the native aboveground biomass, setting the native root biomass to three values. The estimated effects obtained with the package *effects* are shown [1, 2].**


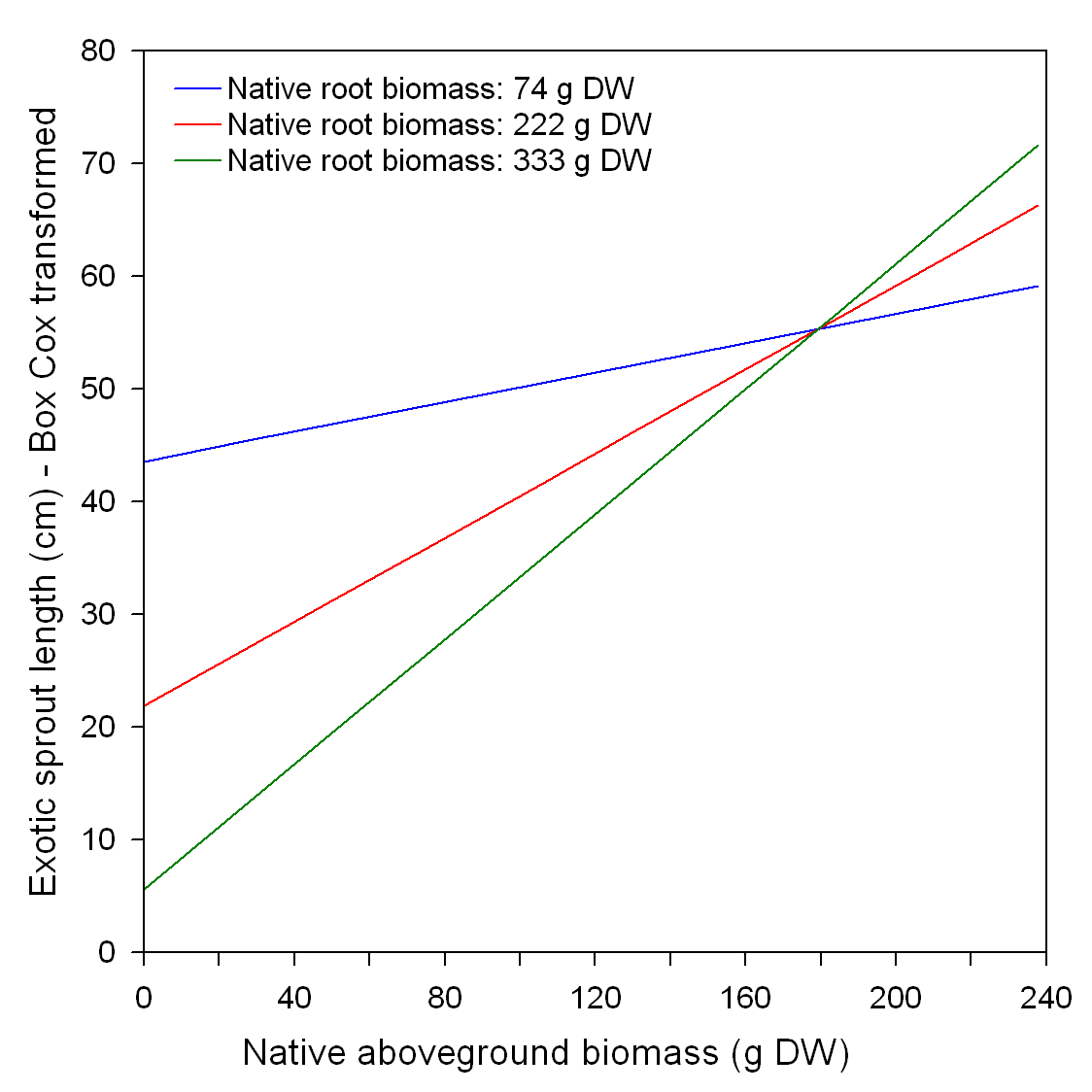


**Reference**

1. Fox J. (2003) Eﬀect Displays in R for Generalised Linear Models. J Stat Softw 8: 1-18.
2. Fox J, et al. (2012) *effects*: Effect Displays for Linear, Generalized Linear, Multinomial-Logit, Proportional-Odds Logit Models and Mixed-Effects Models (R package version 2.2-3). Available at: http://cran.r-project.org/web/packages/effects/.
