# Supplementary material for: Native Macrophyte Density and Richness Affect the Invasiveness of a Tropical Poaceae Species
Source: PLoS One. 2013 Mar 25;8(3):e60004. doi: 10.1371/journal.pone.0060004 (PMC3607602; doi:10.1371/journal.pone.0060004)
Supplement: Figure S3 — Accumulated effect sizes (mean ± CI95%) from Levine et al. (2004) and from our experiment. (DOC) [file pone.0060004.s003.doc]

**Supporting Information**

**Figure S3** – Accumulated effect sizes (mean ± CI_95%_) from Levine *et al*. (2004; see their Fig. 2) and from our experiment.
