# Supplementary material for: Native Macrophyte Density and Richness Affect the Invasiveness of a Tropical Poaceae Species
Source: PLoS One. 2013 Mar 25;8(3):e60004. doi: 10.1371/journal.pone.0060004 (PMC3607602; doi:10.1371/journal.pone.0060004)
Supplement: Appendix S1 — Meta-analysis. (DOC) [file pone.0060004.s004.doc]

**Supporting Information**

**Appendix S1** *Meta-analysis*

The experiments in which we manipulated the native species density can be considered independent studies, allowing comparison of our results with those obtained by Levine *et al*. [1]. These authors used the Hedges statistic [2] to estimate the effect size (*d*) of biotic resistance (the direction of the comparison between groups: the performance of an exotic species with no biotic resistance minus its performance with biotic resistance). Positive values of the *d* statistic indicate a better performance of the exotic species in question (e.g., in terms of biomass, growth rates or germination rates) when grown in isolation (i.e., without the presence of any native species).

To estimate the effect size (*d* statistic), we compared two groups (*Urochloa arrecta* growing alone and in association with natives) using all of the *U. arrecta* biomass data obtained when *U. arrecta* was grown in the presence of a given native species. Differences related to the native species density were therefore ignored to guarantee independence among effect sizes [2]. The biomasses of *U. arrecta* grown in isolation (*n* = 15) were chosen at random to form each group with no biotic resistance, with a *n* = 3 [3].

The accumulated effect size was estimated based on the weighted effect size (*d_++_*). These weights were the reciprocal of the variances of the effect sizes. A fixed-effect model was used for this purpose, considering that the experimental procedure was the same for all species. The significance of the *d_++_* statistic was then assessed based on a confidence interval obtained with bootstrapping [4]. The heterogeneity of the *k* = 5 studies was tested using the *Q* statistic, which follows a χ^2^ distribution with *k*-1 degrees of freedom [2]. Finally, the accumulated effect size estimated in this study was compared with that of Levine *et al*. ([1], see Fig. S3).

We also compared our meta-analytical results with the findings of Balvanera *et al*. [5] and Cardinale *et al*. [6]. Other effect sizes (identical to those estimated in the aforementioned studies) were calculated as defined above. Specifically, a comparison with the results of Balvanera *et al*. [5] was performed by transforming the Pearson correlation coefficients between the native richness and the response variables (*U. arrecta* mean length and total biomass) into the Fisher’s *z* statistic [2], while the comparison with Cardinale *et al*.’s results [6] involved estimating the log response ratio of the data obtained in the experiment in which the native species richness was manipulated. Thus, the proportional difference for each response variable was estimated as , where is the mean value of the response variable derived when *U. arrecta* was grown together with the five native species (maximum richness), and is the mean value when *U. arrecta* was grown in isolation.

**Supporting references**

1. Levine JM, Adler PB, Yelenik SG (2004) A meta-analysis of biotic resistance to exotic plant invasions. Ecol Lett 7: 957-989.

2. Borenstein M, Hedges LV, Higgins JPT, Rothstein HR (2009) Introduction to meta-analysis. West Sussex, John Wiley & Sons.

3. Goldberg DE, Scheiner SM (1993) ANOVA and ANCOVA: Field Competition Experiments. In: Scheiner S M, Gurevitch J (editors) Design and analysis of ecological experiments. Chapman & Hall, New York, pp. 77-98.

4. Rosenberg MS, Adams DC, Gurevitch J (2000) MetaWin: Statistical Software for Meta-analysis. Sinauer, Sunderland, Massachusetts, USA.

5. Balvanera P, Pﬁsterer AB, Buchmann N, He JS, Nakashizuka T, et al. (2006) Quantifying the evidence for biodiversity effects on ecosystem functioning and services. Ecol Lett 9:1146-1156.

6. Cardinale BJ, Srivastava DS, Duffy JE, Wright JP, Downing AL, et al. (2006) Effects of biodiversity on the functioning of trophic groups and ecosystems. Nature 443:989-992.
